# Multiple Metrics of Carbohydrate Quality Place Starchy Vegetables Alongside Non-starchy Vegetables, Legumes, and Whole Fruit

**DOI:** 10.3389/fnut.2022.867378

**Published:** 2022-05-02

**Authors:** Adam Drewnowski, Matthieu Maillot, Florent Vieux

**Affiliations:** ^1^Center for Public Health Nutrition, University of Washington, Seattle, WA, United States; ^2^MS-Nutrition, Faculté de Médecine La Timone, Marseille, France

**Keywords:** Carbohydrate Quality Indices (CQI), Carbohydrate Food Quality Score (CFQS-4), starchy vegetables, white potatoes, legumes, dietary guidelines

## Abstract

**Background:**

Starchy vegetables, including white potatoes, are often categorized as “lower-quality” carbohydrate foods, along with refined grains, 100% fruit juices, sweetened beverages, and sugars, snacks and sweets. Among “higher-quality” carbohydrates are whole grains, non-starchy vegetables, legumes, and whole fruits.

**Objective:**

To apply multiple nutrient profiling (NP) models of carbohydrate quality to foods containing >40% carbohydrate by dry weight in the USDA Food and Nutrient Database for Dietary Studies (FNDDS 2017-18).

**Methods:**

Carbohydrate foods in the FNDDS (*n* = 2423) were screened using four recent Carbohydrate Quality Indices (CQI) and a new Carbohydrate Food Quality Score (CFQS-4). Cereal products containing >25% whole grains by dry weight were classified as whole grain foods.

**Results:**

Based on percent items meeting the criteria for 4 CQI scores, legumes, non-starchy and starchy vegetables, whole fruit, and whole grain foods qualified as “high quality” carbohydrate foods. Distribution of mean CFQS-4 values showed that starchy vegetables, including white potatoes placed closer to non-starchy vegetables and fruit than to candy and soda.

**Conclusion:**

Published *a priori* determinations of carbohydrate quality do not always correspond to published carbohydrate quality metrics. Based on CQI metrics, specifically designed to assess carbohydrate quality, starchy vegetables, including white potatoes, merit a category reassignment and a more prominent place in dietary guidance.

## Introduction

Carbohydrate-rich foods in the global food supply include grains and cereals, legumes, roots and tubers, as well as vegetables and fruit (1). Healthful carbohydrates are a critical component of healthy food patterns (2); however, metrics to evaluate carbohydrate quality are not yet fully established (3). Instead, it appears that decisions about carbohydrate quality are sometimes made *a priori* (4, 5). Treated as “lower quality” carbohydrates in some past studies were starchy vegetables, including white potatoes, refined grains, 100% fruit juices, sweetened beverages, and sugars, snacks, and sweets (4, 5). Assigned to “higher quality” carbohydrates were whole grains, legumes, whole fruit, and non-starchy vegetables, including dark green vegetables and tomatoes (4, 5).

These *a priori* decisions regarding carbohydrate quality may not be consistent with some of the emerging carbohydrate quality indices. Four Carbohydrate Quality Indices (CQI) (3) were recently developed to help identify higher-quality carbohydrate foods, based on carbohydrate-to-fiber and carbohydrate-to-free sugar ratios. Higher quality carbohydrate foods were those with more fiber and less free sugar relative to carbohydrate (3, 6). In one of the CQI models, the ratio of fiber to free sugar was also considered (3, 6). A newly developed CFQS-4 index added sodium, and potassium to fiber and free sugar to assess carbohydrate quality (7). These five methods were used in the present study to assess nutritional density of carbohydrate-rich foods, ranging from grains and cereals to tubers, vegetables, and fruit.

Based on USDA classifications used in What We Eat in America (WWEIA) studies (8), starchy vegetables include white potatoes, sweet potatoes, yams, corn, beans, carrots, beets, turnips, and winter squashes. Non-starchy vegetables include leafy greens, such as cabbage, Brussels sprouts, and lettuce and other salad greens, along with zucchini, peppers, asparagus, and tomatoes. Both types of vegetables provide a wide variety of vitamins and minerals, along with antioxidant flavonoids and other phenolic compounds (9). Among important food sources of dietary potassium are legumes, vegetables, and fruits but also white and sweet potatoes and yams (9, 10). Many starchy vegetables are important sources of dietary fiber (9, 10). Nonetheless, some studies have placed starchy vegetables and white and sweet potatoes alongside sweetened beverages, candy, and sweet bakery goods (4, 5).

Nutrient profiling models are routinely used to assess the relative healthfulness of different foods (11, 12). There are ways to assess the quality of selected nutrients such as protein (13). *A priori* determinations of carbohydrate quality ought to be cross-checked against existing CQI scores that were developed specifically for that purpose (3).

The present analyses used USDA food category codes to assign foods into grains, snacks and sweets, vegetables, legumes, fruits, and beverages (8). Nutrient density of carbohydrate-rich foods was assessed using the publicly available Food and Nutrient Database for Dietary Studies (FNDDS 2017-18) (14). The testing algorithms included four published CQI scores (3) and a newly developed carbohydrate food quality score (CFQS-4).

## Materials and Methods

### The What We Eat in America Coding Schemes

Nutrient composition data for carbohydrate foods came from the USDA Food and Nutrient Database for Dietary Studies (FNDDS 2017-2018) that is available online (14). The FNDDS 2017-18 database lists 7,083 items, aggregated using What We Eat in America (WWEIA) coding schemes (8). One-digit codes identify 9 major food groups: grains, snacks and sweets, beans and nuts, vegetables, fruits, milk, meat, eggs, and fats and oils. Two-digit WWEIA codes identify 53 smaller food subgroups. For example, foods in the grains group are separated into cooked grains, breads, quick breads, ready-to-eat cereals (RTE), and cooked cereals. Four-digit WWEIA codes identify 138 food categories. For example, sweet bakery products, coded under snacks and sweets are now separated into cakes and pies, cookies and brownies, and donuts, pastries and sweet rolls. The eight-digit WWEIA codes correspond to individual foods. The FNDDS 2017-18 was merged with the Food Patterns Equivalents Database (FPED) to obtain whole grain content of foods. Free sugars were defined as added sugars, sugars from 100% fruit juice, sugars in sweetened beverages, jams and jellies, and honey, sugars and syrups.

### Selecting Carbohydrate Foods From Food and Nutrient Database for Dietary Studies 2017-18

Only foods with ≥40% energy from carbohydrate per 100 g dry weight were included in the present analyses. Food dry weight and carbohydrate dry weight were calculated as derived variables. Non-starchy vegetables, legumes and fruits were identified using WWEIA classification codes. Whole grain content of foods was obtained from the FPEDs database and converted to g/100 g (15, 16). Following past work, whole grain CF were identified as those foods that contained > 25% whole grain by dry weight (15, 16). Current US regulations state that foods containing at least 25% whole grain by dry weight may make a front of pack claim. For foods to be called whole grain in the product name, at least 50% by dry weight is required. CF with > 25% whole grain dry weight (*n* = 337) were removed from the grains, snacks and sweets groups and placed in a separate whole grains category.

Following past studies (4, 5), refined grains, snacks and sweets, 100% fruit juices, sweetened beverages, and starchy vegetables were defined as “lower quality” CF. Based on WWEIA codes, refined grains were cooked grains, breads and rolls, quick breads, RTE cereals, and cooked cereals. Snacks and sweets were savory snacks, crackers, snack/meal bars, sweet bakery products, candy, other desserts, sugars and sweetened beverages. Starchy vegetables included white and sweet potatoes, corn, yams and winter squash. The present analyses included 100% fruit juices and sugar-sweetened beverages (carbonated and not), since those food groups had been assigned to the “lower quality” CF category in previous studies (4, 5). Diet beverages and foods with energy density <10 kcal/100 g were excluded. Meats, milk, and dairy products were not viewed as primary carbohydrate sources and were excluded. Also excluded were baby foods and infant formula, non-reconstituted nutrition powders, and items not classified as foods. The analytical database was composed of 2423 foods. The food categories are listed in Table 1.

**TABLE 1:** Carbohydrate foods (CF) in the FNDDS 2017-18 database classified by What We Eat in America 4-digit codes and by “high quality and “low quality” assignments in refs (4, 5).

Food categories	Food subcategories	WWEIA 4-digit code	N	Whole grain
“**High quality” CF**				
Legumes	Beans, peas, soy	2802, 2806	76	
Non-starchy vegetables	Dark green leafy, green, red, and orange	6402–6414, 6420–6489	351	
Fruits	Fresh, canned, frozen, dried	6002–6024	117	
Whole grains	CF with > 25% WG by dry weight	See below	337	>25% WG
**“Low quality” CF**				
Refined grains	Cooked grains	4002, 4004	29	EXCEPT those with > 25% WG by dry weight
	Breads, rolls, tortillas	4202–4208	133	
	Quick breads	4402, 4404	99	
	RTE cereals	4602, 4604	37	
	Cooked cereals	4802, 4804	62	
Snacks and sweets	Savory snacks	5002–5008	97	
	Crackers	5202, 5204	42	
	Snack/meal bars	5402, 5404	41	
	Sweet bakery	5502, 5504, 5506,	352	
	Candy	5702, 5704	130	
	Other desserts	5802, 5804, 5806	105	
	Sweetened beverages	7202, 7204, 7206, 7208, 7220	114	
	Sugars	8802, 8806	45	
100% Fruit juice	100% Fruit juice (e.g., apple juice, citrus juice)	7002, 7004, 7006, 7008	46	
Starchy vegetables	Starchy vegetables (e.g., corn, potatoes)	6416, 6418, 6802, 6804, 6806	210	
Total			2423	

### Carbohydrate Quality Indices

Early models for assessing carbohydrate quality (17, 18) were based on the carbohydrate-to-fiber ratios. Liu et al. (3) expanded the original 10:1 carbohydrate:fiber model by including free sugars, also in the 10:1 ratio. Higher quality carbohydrate foods were those with > 10% of dietary fiber and < 10%g of free sugars per 100 g of carbohydrate. The 10:1:1 model was justified with reference to recommended intakes of 50% of energy from carbohydrates but only 5% from free sugars (3). The accompanying 10:1:2 model maintained the 10:1 carbohydrate:fiber ratio but relaxed the free sugar threshold to 20% of free sugars per 100 g of carbohydrate (3). The final 10:1|2:1 model required the 10:1 carbohydrate:fiber ratio and <2 g of sugar per 1 g of fiber (<2:1) (3). The performance of these four CQI scores, applied to largely processed carbohydrate foods, is described elsewhere (3). The models are summarized in Table 2.

**TABLE 2:** Selected indices of carbohydrate quality.

Index	Model	Description of elements	Range
Single point scores	10:1f	>1 g of fiber per 10 g of carb	0 to 1
	10:1s	<1 g of free sugars per 10 g of carb	0 to 1
	10:2s	<2 g of free sugars per 10 g of carb	0 to 1
	2:1s	<2 g of free sugars per 1 g of fiber	0 to 1
CQI models	10:1:1	combined 10:1f + 10:1s	0 to 2
	10:1:2	combined 10:1f + 10:2s	0 to 2
	10:1|2:1	combined 10:1f + 2:1s	0 to 2
CFQS-4	FSNaK	10:1f + 10:1s + sodium + potassium	0 to 4

*Models from refs (3, 7).*

### Carbohydrate Food Quality Score

The purpose of NP models is to assist in the construction of healthy food patterns consistent with dietary guidelines (9). The 2020-25 DGA recommend increasing whole grains, fiber, and potassium while reducing both sugar and sodium (9). These five components are directly relevant to assessing the healthfulness of carbohydrate foods. The current guidelines for sodium reduction issued by the US Food and Drug Administration specifically address the need to reduce sodium in processed carbohydrate foods (19). Potassium, a shortfall nutrient is the US diet is now required to be listed on the back-of-pack Nutrition Facts Panel (20). The point system for CFQS-4 score built on the same 10:1 ratios for fiber and free sugars relative to carbohydrate (3), but with points added for high potassium and low sodium content of foods (see Table 2). The cutoff level for sodium was based on <600 mg sodium/100 dry weight. The cutoff for potassium was > 300 mg/100 g dry weight. These values roughly correspond to median values for each nutrient in the FNDDS.

### Statistical Analyses

The distribution of carbohydrate foods among food groups and food subgroups was shown using pie charts. Frequency and percentage of foods that met the CQI criteria were calculated for each food group. Kappa coefficients were calculated between all scores. Means of energy density and carbohydrate quality scores were calculated food groups and subgroups. Scatterplots were used to compare energy and carbohydrate quality scores across food groups. A *p*-value < 0.05 was considered as significant. All statistical analysis were conducted with SPSS (Statistical Package for the Social Sciences, IBM).

## Results

### Carbohydrate Foods in the Food and Nutrient Database for Dietary Studies 2017-18

**FIGURE 1:**
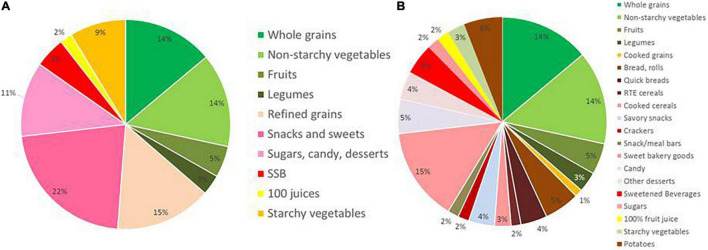
Carbohydratefood distribution by What We Eat in America (WWEIA) food subgroup **(A)**. Carbohydrate food distribution by WWEIA food category **(B)**.

The present analytical sample was 2423 foods from FNDDS 2017-18. These foods were selected using What We Eat in America 4-digit codes and included grains, snacks and sweets, sugar sweetened beverages, 100% fruit juices, vegetables, and fruit. Of these, 36% were pre-classified as “higher quality” and 64% as “lower quality” carbohydrates following published papers (4, 5). Figure 1A shows the distribution of FNNDS carbohydrate foods by WWEIA food subgroup. Figure 1B shows the same distribution by WWEIA food category.

### Carbohydrate Quality Index Values

The present goal was to compare published assignments of CF quality (4, 5) against 4 published carbohydrate quality index (CQI) metrics (3). Table 3 shows how many “higher” and “lower” quality carbohydrates met the published CQI criteria. Shown are numbers and percentages for each food group (3). Among these foods, most did not meet any of the CQI metrics. The highest number (35.5%) met the 10:1 CQI (35.5%), followed by the 10:1:2 (30.7%), 10:1:1 (28.5%) and 10:1|2:1 (28.1%) Those percentages are higher than those reported by Liu et al. (3) because high-scoring and carbohydrate-rich legumes, vegetables and fruit were included in the present sample. Highest percentages of “high quality” CF (based on the 4 CQI metrics) were obtained for legumes, non-starchy vegetables, fruit, starchy vegetables, and whole grains.

**TABLE 3:** Comparison of carbohydrate-rich foods in the US meeting each of four metrics for assessing carbohydrate quality*^a^*.

Food categories^b^		Number and percent foods meeting each CQI metric^c^										
		
		10:1 (1 point)		10:1:1 (2 points)		10:1:2 (2 points)		10:1|2:1 (2 points)		Kappa^d^ 10:1 & 10:1:1	Kappa^d^ 10:1 & 10:1:2	Kappa^d^ 10:1 & 10:1|1:2
								
	N	N	%	N	%	N	%	N	%			
All foods	2423	860	35.5	690	28.5	743	30.7	681	28.1	0.84	0.89	0.83
**“High quality” carbs**												
Non-starchy vegs	351	323	92.0	311	88.6	313	89.2	311	88.6	0.805	0.833	0.805
Legumes	76	72	94.7	67	88.2	68	89.5	67	88.2	0.585	0.642	0.585
Fruits	117	73	62.4	62	53.0	67	57.3	59	50.4	0.809	0.894	0.760
Whole grains	337	183	54.3	109	32.3	146	43.3	103	30.6	0.574	0.783	0.541
**“Low quality” carbs**												
Refined grains	360	44	12.2	37	10.3	42	11.7	37	10.3	0.903	0.974	0.903
Snacks and sweets	532	28	5.3	8	1.5	8	1.5	8	1.5	0.431	0.431	0.431
Sugar, candy, desserts	280	15	5.4	1	0.4	2	0.7	1	0.3	0.119	0.226	0.119
Sweetened beverages	114	16	14.0	0	0.0	0	0.0	0	0	0	0	0
100% fruit juice	46	9	19.6	0	0.0	0	0.0	0	0	0	0	0
Starchy vegetables	210	97	46.2	95	45.2	97	46.2	95	45.2	0.981	1.00	0.981

*^a^We used data from the Food and Nutrient Datanase for Dietary Studies 2017-18 to identify carbohydrate-rich foods in the US diet.*

*^b^Products were aggregated into 12 food categories based on the WWEIA food categories. The whole grain category was composed of foods containing > 25% whole grains by dry weight.*

*^c^The proposed carbohydrate quality metrics were based on Liu et al. (3) and were based on per 100 g of carb:(a) 10 g fiber (10:1 carb:fiber), (b) 10 g fiber and <10 g free sugars (10:1:1 carb:fiber:free sugars), (c) 10 g fiber and <20 g free sugars (10:1:2 carb:fiber:free sugars); and (d) 10 g fiber and, per each 10 g of fiber, <20 g free sugars (10:1 carb:fiber, 1:2:fiber:free sugars). Values represent number (#) of products and percent (%) of foods in each food group meeting each of the criteria.*

*^d^Kappa is a measure of agreement between the metrics. 0 indicates the agreement is by chance; 0.01–0.20 indicates slight agreement; 0.21–0.40 indicates fair agreement; 0.41–0.60 indicates moderate agreement; 0.61–0.80 indicates substantial agreement; 0.81–0.99 indicates almost perfect agreement.*

Considerable variation was observed across different food categories. Nearly all non-starchy vegetables and legumes and most fruit contained > 10 g of fiber per 100 g of carbohydrate and scored a point in the 10:1 CQI. So did most starchy vegetables that were slightly below legumes but well above fruits. Foods with lower fiber content were primarily snacks and sweets, sugar, candy and desserts and refined grains. Some 100% fruit juices did have adequate fiber to carbohydrate ratios and also scored a point in CQI 10:1. By contrast, only 5.3 of snacks and sweets met the 10:1 CQI.

The 10:1:1 model based on fiber and free sugar, was more restrictive, consistent with past observations (3). Sugars, sweetened beverages, candy, and desserts did not pass the free sugar threshold. Although the 10:1:2 CQI score used a more relaxed free sugar threshold compared to 10:1:1 (3), the two models were not very different from each other. Following Liu et al. (3), kappa statistics were used to assess the degree of association among the CQI models.

For all foods, agreement across the four CQI metrics was very good, as shown in Table 3 (3). The lowest agreement was between the fiber ratio model 10:1 and the other three models that included both free sugar and fiber (kappa ≥ 0.80). Kappa values were 0.84 for 10:1 and 10:1:1 and 0.83 for 10:1 and 10:1|1:2. The other three models showed almost perfect agreement with kappa ranging from 0.95 to 0.99.

Agreement across the metrics was highly variable, as reported previously (3). Agreement was very high (kappa > 0.8) for refined grains, starchy vegetables, fruit and non-starchy vegetables. Agreement was lowest (kappa < 0.25) for sugar, candy, and desserts.

**FIGURE 2:**
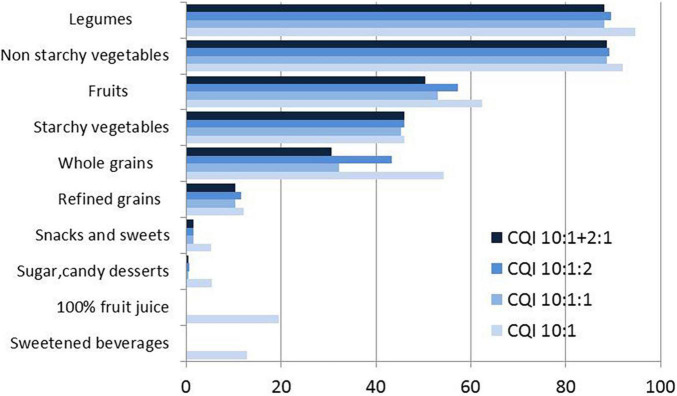
Percent assignment to high quality CF based on four CQI models (3) by food group. The three models are: A) CQI 10:1, B) CQI 10 10:1:1, C) CQI 10:1:2, and D) CQI 10:1}1:2.

Percentages of items within each food category that could be classified as “higher quality” carbohydrates based on the 4 CQI metrics (3, 6) are also shown in Figure 2. Legumes had the highest percent of foods meeting CQI criteria, followed by fruits and starchy vegetables. Only 10% of refined grains met the CQI criteria for high quality carbohydrates. Sweetened beverages, 100% fruit juices, sugar, candy, desserts, and sweets did not meet CQI criteria.

**FIGURE 3:**
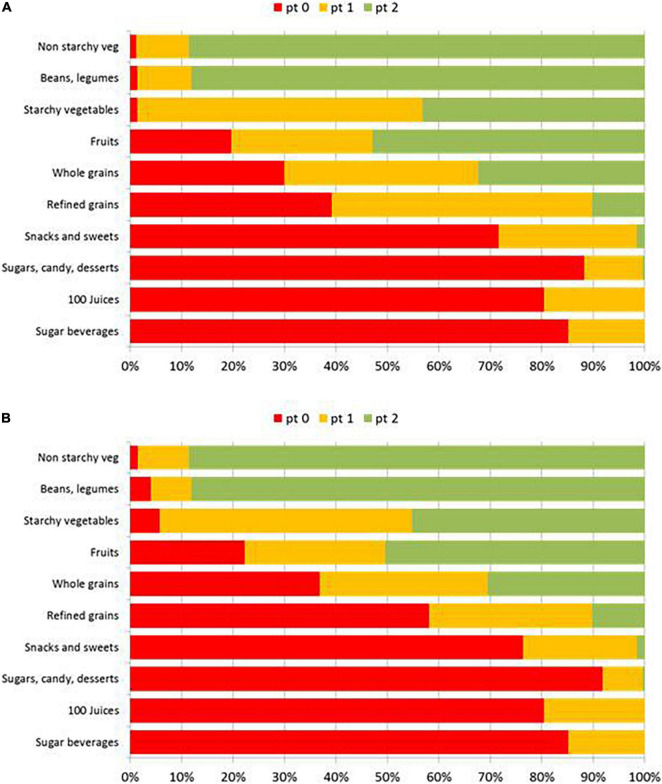
Distribution of points scores for the 10:1:1 model **(A)** and the 10:1|2:1 model by **(B)** WWEIA food subgroup.

The present analyses point to a discrepancy between *a priori* assignments of CF quality (4, 5) and CF quality metrics. Based on the percent of items meeting CQI criteria, starchy vegetables were more likely to meet those criteria compared to refined grains, snacks and sweets, candy and desserts, and sweet beverages. Figure 3 shows the distribution of point scores for the 10:1:1 model and the 10:1|2:1 model by WWEIA food subgroup. Based on published CQI scores, it would appear that starchy vegetables do not belong together with sweetened beverages, sweets, candy and other desserts.

**FIGURE 4:**
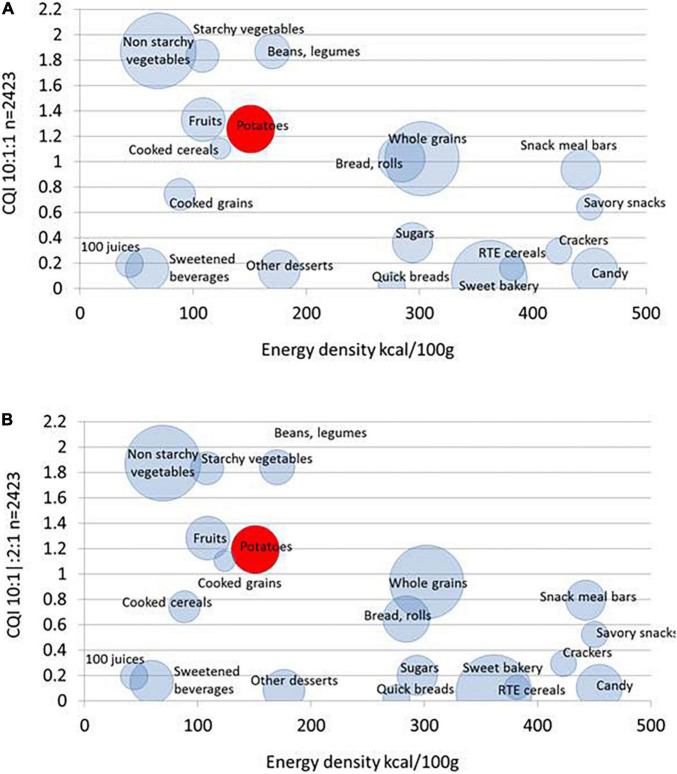
Scatterplot of CQI 10:1:1 model scores **(A)** and CQI 10:1|2:1 model scores **(B)** plotted against energy density by WWEIA food category. The size of the bubble represents the number of items in the category.

Figure 4A is a scatterplot of mean point scores from the CQI 10:1:1 model plotted against energy density by more granular WWEIA food category. Figure 4B is a scatterplot of mean point scores from the CQI 10:1—2:1 model plotted against energy density by WWEIA food category. Size of the bubble corresponds to number of foods in each category.

Although the two models were based on different fiber to free sugar ratios, both CQI metrics produced similar results. First, mean CQI scores for non-starchy vegetables and starchy vegetables were the same. Non-starchy vegetables are conventionally assigned to high quality CF whereas starchy vegetables are assigned to low quality CF. Second, mean CQI 10:1:1 ratings for potatoes were similar to those for whole fruit (fried potatoes had higher energy density). Mean CQI ratings for potatoes were comparable to those for cooked cereals (those with < 25% whole grains). In all cases, starchy vegetables including potatoes were far removed from candy, sweet bakery goods, sweetened beverages, sugars, snacks and sweets. Rather, two CQI metrics placed starchy vegetables among higher-quality carbohydrate-rich foods.

### Carbohydrate Food Quality Score Values

Figure 4 is a scatterplot of energy density and another recently developed carbohydrate food quality score, CFQS-4, that is based on fiber, free sugar, potassium, and sodium. The scatterplot shows WWEIA food subgroups, with potatoes are now separated into boiled, mashed, and fried. The size of bubble represents the number of items in that WWEIA subgroup in FNDDS.

First there was a clear separation of different food subgroups by energy density. Energy density is a crude measure of nutritional value. Energy dense candy snacks and sweets were on the right; lower energy vegetables (starchy and not), fruit, and legumes were on the left. However, energy density does not fully capture nutritional value; sugary beverages with low energy density received very low CFQS-4 scores.

Figure 4 shows that mean energy density and CFQS-4 values for non-starchy vegetables, starchy vegetables, fruit and beans and legumes were very close. Boiled potatoes had the highest CFQS-4 values followed by fried and mashed potatoes. What is apparent is that white potatoes have energy density and CFQS-4 values that are distinct from those for most refined grains, candy, snacks, and sweets.

**FIGURE 5:**
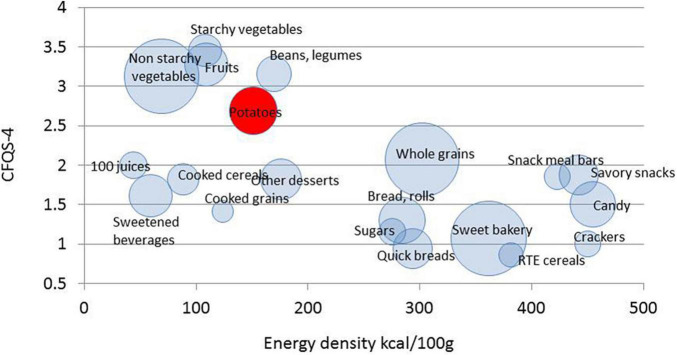
Scatterplot of mean CFQS-4 scores plotted against energy density (kcal/100) by WWEAI food subgroup. The size of bubble represents the number of items in that subgroup.

Figure 5 also shows that the distribution of CFQS-4 scores places potatoes closer to high scoring carbohydrates (points 3–4 were collapsed) than to candy and soda.

## Discussion

High-quality carbohydrate foods are vital components of healthy diets (21); however, as noted in a recent paper (3), metrics to define carbohydrate quality are not as yet well-established. Such scores of carbohydrate quality would be directly relevant to dietary guidance. Increasing dietary fiber and reducing free sugars was a component of the Dietary Guidelines for Americans 2020-25 (8). Both those elements have been incorporated into CQI metrics. Ratio-based measures of carbohydrate quality based on > 10% fiber content per 100 g carbohydrate (10:1 carb/fiber ratio) have been in place for several years (21–26). The more recent CQI added a free sugar component (<10% or <20% per 100 g carbohydrate), defining high-quality CF as those with higher fiber and lower free sugar content (3).

These new CQI scores permit a re-evaluation of some past judgments of carbohydrate quality that have appeared in the literature (4, 5). In some recent studies, starchy vegetables, including white potatoes were classified as “low quality” carbohydrates, along with refined grains, 100% fruit juices, sweetened beverages, and sugars, snacks, and sweets (4, 5). Our analytical sample of 2423 items differed from the 2,208 carbohydrate-rich products of Liu et al. (3). In addition to previously tested grains, cereals, candy, snacks and sweets, the present sample included vegetables, fruit, legumes, 100% juices and sweetened beverages. All those food groups had been assigned into different categories of carbohydrate quality by other authors (4, 5) but had not been screened using published or new CQI metrics (3, 6).

It would appear from the present analyses that the new CQI metrics (3) do not support some former judgments of carbohydrate quality (4, 5). In particular, the four CQI metrics (3) gave similar ratings to starchy and to non-starchy vegetables. Ratings for white potatoes were higher for boiled than for mashed or fried potatoes. Even so, white potatoes were closer in terms of CQI scores to starchy vegetables and to legumes than to candy and sweets. Our analytical sample went beyond processed grain-based foods by including diverse food groups that had previously been assigned to “high” and “low” quality carbohydrate categories.

The reported pass rate for the 10:1 ratio model was 23.2% of processed CF; while the 10:1:1 model was more restrictive and passed only 16.4%, consistent with other reports (3). Our sample had vegetables, legumes and fruit (but also juices and SSB). The free sugar component (which includes added sugars, as well as sugars from jams, jellies, honey, and syrups) was responsible for assigning candy, sweet bakery goods, and other desserts to the lower quality carb category, without affecting cooked grains, vegetables, beans and legumes, and vegetables. The pass rate for those food groups was accordingly much higher. The question arises whether some of the existing CQI metrics are sufficiently inclusive to serve as measures of carbohydrate food quality overall or are they best applied to the narrower subsample of processed grain foods.

The new CFQS-4 model (7) added sodium and potassium to the 10:1:1 model (3) to better align with DGA recommendations. Most CF quality metrics have not yet included these important elements. Yet sodium reduction in processed foods, including grain-based foods, is a priority for both the DGA and the FDA (8, 19). Second, potassium content of foods is required to be listed on FDA-approved food labels (20). One characteristic of most “high quality” fruits, vegetables, beans and legumes is their low sodium and high potassium content. It is worth noting that 100% fruit juices contain free sugar but are also important sources of dietary potassium. Nutrient density models strive to capture the overall nutritional value of foods.

As expected, most vegetables (starchy and not), legumes and fruit were assigned to higher-quality carbohydrates by the 4 CQI models and by CFQS-4. Starchy vegetables tend to be high in both potassium and fiber and low in free sugars and sodium (27, 28). Based on the present results, it may be time to place starchy vegetables among the higher-quality CFs.

Whole grains are another important index of carbohydrate quality (21). In the present sample, the whole grain category was drawn primarily from breads, cooked cereals, cooked grains, RTE cereals, and savory snacks. However, whole grains are not the only index of carbohydrate quality; whole grain bakery goods can contain both added sugar and sodium. As a result, whole grain foods performed less well than expected on the Liu et al. CFQ models. This brings up the issue of how to reconcile ingredient-based classification of carbohydrate quality (4, 5) and purely nutrient-based quality standards (3) when applied to the same foods. Hybrid nutrient density scores that incorporate both nutrients and selected ingredients may provide the needed answer (29, 30). One such score does incorporate whole grains alongside some key nutrients to arrive at a total nutrient density score (29, 30).

One general question is whether NP models truly capture nutrient density of foods. A second question is whether nutrient-based models can be applied to evaluate the quality of a macronutrient (carbohydrate, protein, fat). The present analyses were limited to selected food groups and only those foods that contained > 40 carbohydrate by dry weight. There is also the question whether NP models need to incorporate such elements as bioavailability, fortification, or food matrix effects. As dietary guidance becomes more food based (31) and more concerned with sustainability, NP models need to evolve as well (29, 30).

The present indices of carbohydrate quality were relatively simple and based on selected nutrients only. No model included vitamins or minerals other than potassium and sodium. These components are parts of other NP models, both category specific and across the board, that were designed to capture the nutritional value of all foods. Second, none of the models used here included the glycemic index, partly because of its limitations (32, 33). At this time, GI values are not recognized as a food quality metric and the relevant values are not available in publicly funded databases. These elements could be integrated in future carbohydrate food quality metrics.

## Conclusion

Practical CQI metrics were specifically developed to help identify high quality carbohydrate rich foods. These metrics, when applied to the FNDDS WWEIA food groups challenge popular assumptions. The tendency has been to separate starchy vegetables (including potatoes) from non-starchy vegetables and categorize them alongside candy, sugary beverages, snacks, and other desserts. Yet there is evidence that the intake of potatoes is associated with higher diet quality and higher nutrient adequacy among adolescent in the US (34). This research report advances this work by applying multiple NP models to WWEIA foods that were classified as low- and high-quality CF. The present analyses based on published CQI metrics make a case for a category reassignment and an affirmation that all vegetables do in fact belong together.

## Data Availability Statement

Publicly available datasets were analyzed in this study. This data can be found here: all data for this project are publicly available on the United States Department of Agriculture Website, available at: https://www.ars.usda.gov/northeast-area/beltsville-md-bhnrc/beltsville-human-nutrition-research-center/food-surveys-research-group/docs/fped-overview/.

## Author Contributions

AD, MM, and FV collaborated on the intellectual conception, design, and development of this manuscript. AD drafted the original manuscript. All authors contributed to the writing and editing of the manuscript and approved the final manuscript.

## Conflict of Interest

AD is invited member of the Quality Carbohydrate Coalition’s Scientific Advisory Council (QCC-SAC). AD is the developer of the Nutrient Rich Food (NRF) index, a nutrient profiling model, and has received grants, contracts, and honoraria from entities, both public and private, with an interest in nutrient density of foods, complex meals, and the total diet. MM and FV are with MS-Nutrition, a nutrition analysis start-up, located in Marseille, France.

## Publisher’s Note

All claims expressed in this article are solely those of the authors and do not necessarily represent those of their affiliated organizations, or those of the publisher, the editors and the reviewers. Any product that may be evaluated in this article, or claim that may be made by its manufacturer, is not guaranteed or endorsed by the publisher.
